# Health and performance effects of 12 weeks of small-sided street football training compared to grass football training in habitually active young men

**DOI:** 10.1007/s00421-023-05308-y

**Published:** 2023-09-15

**Authors:** Morten B. Randers, Marie Hagman, Jesper F. Christensen, Susana Póvoas, Jens Jung Nielsen, Peter Krustrup

**Affiliations:** 1 Department of Sports Science and Clinical Biomechanics, SDU Sport and Health Sciences Cluster (SHSC), University of Southern Denmark, Campusvej 55, 5230 Odense, Denmark; 2 School of Sport Sciences, Faculty of Health Sciences, UiT The Arctic University of Norway, Tromsø, Norway; 3 Centre for Physical Activity Research, Rigshospitalet, University of Copenhagen, Copenhagen, Denmark; 4 The Department of Sports Science and Clinical Biomechanics, Faculty of Health Sciences at the University of Southern Denmark, Odense, Denmark; 5 Digestive Disease Center, Bispebjerg Hospital, Copenhagen, Denmark; 6 Research Center in Sports Sciences, Health Sciences and Human Development, CIDESD, University of Maia, Maia, Portugal; 7 Sport of Nutrition, Exercise and Sports, University of Copenhagen, Copenhagen, Denmark; 8 Sport and Health Sciences, University of Exeter, Exeter, UK; 9 Danish Institute for Advanced Study (DIAS), Faculty of Health Sciences, University of Southern Denmark, Odense, Denmark

**Keywords:** Small-sided games, Soccer, VO_2_max, Body composition, Game formats

## Abstract

**Purpose:**

The purpose of the present study was to investigate the health and exercise performance effects of street football training on very small pitches surrounded by boards in young habitually active men in comparison to small-sided football training on grass.

**Methods:**

Thirty-nine habitually active men (30.7 ± 6.7 years, 90.9 ± 16.6 kg, 183.8 ± 4.5 cm, 39.6 ± 6.0 mL/min/kg) were randomly assigned to a street football training group (ST) or grass football group (GR) playing small-sided games for 70 min, 1.5 and 1.7 times per week for 12 weeks, respectively, or an inactive control group (CO). Intensity during training was measured using heart rate (HR) and GPS units. Pre- and post-intervention, a test battery was completed.

**Results:**

Mean HR (87.1 ± 5.0 vs. 84.0 ± 5.3%HRmax; *P* > 0.05) and percentage of training time above 90%HRmax (44 ± 28 vs. 34 ± 24%; *P* > 0.05) were not different between ST and GR. VO_2_max increased (*P* < 0.001) by 3.6[95% CI 1.8;5.4]mL/min/kg in GR with no significant change in ST or CO. HR during running at 8 km/h decreased (*P* < 0.001) by 14[10;17]bpm in ST and by 12[6;19]bpm in GR, with no change in CO. No changes were observed in blood pressure, resting HR, total body mass, lean body mass, whole-body bone mineral density, fasting blood glucose, HbA1c, plasma insulin, total cholesterol(C), LDL-C or HDL-C. Moreover, no changes were observed in Yo-Yo IE2 performance, 30-m sprint time, jump length or postural balance.

**Conclusion:**

Small-sided street football training for 12 weeks with 1–2 weekly sessions led to improvements in submaximal exercise capacity only, whereas recreational grass football training confirmed previous positive effects on submaximal exercise capacity as well as cardiorespiratory fitness.

## Introduction

Physical activity is essential in the prevention of non-communicable diseases (Pedersen and Saltin 2015). In recent years, many studies have shown that recreational team sports and especially football are very effective in improving participants’ health profile as these sports provide broad-spectrum training stimuli (Hunt et al. 2014; Krustrup et al. 2018a, b; Castagna et al. 2020; Milanović et al. 2019). Recreational football elicits high heart rates (HR) and high level of activity irrespective of age, gender, social background, prior football experience and number of players (Randers et al. 2010b, 2014a, 2018b). Furthermore, recreational football interventions to improve health can easily be organised (Krustrup et al. 2018b; Brito et al. 2018).

In most of the previous training studies on recreational football, grass pitches 20–50 m wide and 40–65 m long have been used, but in larger cities access to grass pitches can be a limitation (Krustrup et al. 2009, 2010a; Randers et al. 2010a; Krustrup and Bangsbo 2015; Sarmento et al. 2020). As urban areas undergo renewal, several street football pitches or football cages, i.e., pitches surrounded by boards and/or a net to keep the ball in play, are being built. Playing small-sided football with boards compared with no boards leads to higher HR and plasma ammonia as well as a higher number of accelerations and rating of perceived exertion, whereas lower total running distance, number of high-speed runs and peak speed are observed (Randers et al. 2020). Thus, even though the distance covered in total and with high-speed running were lower during street football with boards, the high impact on musculoskeletal and cardiovascular systems may lead to comparable improvements in health profile as observed in previous studies using small-sided football played on grass pitches (Barbosa et al. 2021; Milanović et al. 2019; Krustrup et al. 2018a; Sarmento et al. 2020). Until now, the health effects of street football have only been studied in combination with fitness centre training in homeless and socially deprived men with very heterogeneous health profiles (Randers et al. 2012; Helge et al. 2014), showing positive broad-spectrum health improvements after 12 weeks of training.

Thus, the purpose of the present study was to investigate the health and exercise performance effects of small-sided street football training on very small pitches surrounded by boards in young habitually trained men in comparison to small-sided football training on grass. We hypothesized that small-sided street football would be as effective in eliciting high exercise intensities and in improving maximal oxygen uptake, submaximal exercise capacity, lowering blood pressure and resting HR as well as altering body composition and metabolic health as small-sided grass football.

## Methods

### Participants

For recruitment purposes, 51 men were tested, of which 39 (age: 30.7 ± 6.7 years, body mass: 90.9 ± 16.6 kg, height: 183.8 ± 4.5 cm, VO_2_max: 39.6 ± 6.0 mL/min/kg) met the inclusion criteria being non-smoking, no regular participation in organised sport within the last 12 months prior to the study and having a VO_2max_ of 35–48 mL/min/kg. All participants were fully informed about the study and possible risks before giving their written consent to participate. The study followed the Declaration of Helsinki and was approved by the Ethics Committee of Copenhagen (H-1-2013-80).

After baseline testing, the participants were paired according to their VO_2_max and were randomly assigned to either street football training (ST, *n* = 13) or football training on grass (GR, *n* = 13) for 12 weeks. In addition, an inactive control group (CO, *n* = 13) was recruited. In total, 9 players dropped out of the study equally distributed between the three groups (3 due to minor injuries, 1 from each group, and 6 for personal reasons), ending with *n* = 10 in each group. The total number of training sessions completed during the 12-week period were 18.2 ± 6.3 (1.5 ± 0.5 times per week) in ST and 20.9 ± 6.9 (1.7 ± 0.5 times per week) in GR.

### Training intervention

Football training was offered 3 times per week, and participants were encouraged to attend at least 2 out of these 3 sessions. Each training session lasted 70 min and consisted of a short (5–10 min) warm-up based on FIFA-11+followed by 4 × 12 min football games interspersed with ~ 3 min breaks. The street football group played either 2v2 or 3v3 on an 18.5 × 10 m (area per player: 31–46 m^2^) pitch surrounded by boards and net to keep the ball in play all the time. The goals were 3 m wide and 1.6 m high. The grass football group played 2v2 to 5v5 on a 25–40 m long and 15–25 m wide (area per player: ~ 100 m^2^) grass pitch, with 3 m wide and 1.5 m high goals. For both groups, the time spent at the goalkeeper position was equally distributed between the participants within each training session.

### Test procedures

Before and after the 12-week training period, the participants completed a test battery. No training or other intense physical activity were performed during the last 48 h prior to testing.

Participants completed a treadmill test consisting of 6 min walking at 6.5 km/h and 6 min running at 8 km/h interspersed with and followed by a 2-min break before starting an incremental test to exhaustion. The incremental test started with 4 min at 8 km/h before increasing the speed 1 km/h every minute. During the entire test, pulmonary oxygen uptake was measured by a gas analyser (OxyconPro, VIASYS Healthcare, Hoechberg, Germany) and heart rate (HR) was monitored using a Polar HR monitor (Polar Team2 System, Polar Electro Oy, Kempele, Finland). Prior to the test, after the 6-min walking bout at 6.5 km/h, after the 6-min running bout at 8.0 km/h, and at exhaustion, blood lactate was collected from a catheter in the antecubital vein and analysed using ABL 800 flex (Radiometer, Copenhagen, Denmark).

On a separate day, participants arrived in the laboratory after an overnight fast and completed a whole-body Dual-energy X-ray Absorptiometry (LUNAR, GE Medical Systems, Madison, WI, USA). After at least 10 min of rest in the supine position in a quiet room, blood pressure was measured (Omron M7, Omron Healthcare, Kyoto, Japan) from the left upper arm. A mean value was calculated from 6 measurements separated by 1 min. During the entire rest, an HR monitor (Polar Team2 System, Polar Electro Oy, Kempele, Finland) was worn to measure resting HR, defined as the lowest value registered. After these measurements, a blood sample was drawn from an antecubital vein and analysed for fasting blood glucose, plasma insulin, glycosylated haemoglobin (HbA1c), total cholesterol, low-density lipoprotein cholesterol (LDL-C), high-density lipoprotein cholesterol (HDL-C) and triglycerides (Cobas, LiA, Rigshospitalet, Copenhagen, Denmark).

A battery of exercise performance tests including postural balance, jumping, sprint and intermittent high-intensity running capacity were completed on a separate day. After a proper standardised warm-up, postural balance was tested by the Flamingo Balance test (Eurofit Testing Battery, 1988). Standing long jump without the use of arms was measured. Sprint performance was measured using a 30-m sprint test using photocells (Witty system, Microgate, Bolzano, Italy). Intermittent high-intensity running capacity was tested using the Yo-Yo intermittent endurance level 2 test (Yo-Yo IE2) until voluntary exhaustion (Krustrup et al. 2015).

### Heart rate monitoring and time-motion characteristics

HR was recorded at 1-s intervals using Polar HR monitors (Polar Team2 System, Polar Electro Oy, Kempele, Finland) during testing and during randomly selected training sessions encompassing all participants, at the beginning, middle, and at the end of the intervention period. HR is presented relative to the individual maximal HR obtained as the highest HR measured during the incremental treadmill test to exhaustion, the Yo-Yo IE2 or during the training sessions (Póvoas et al. 2019). To describe the training sessions' demands, only data from the playing periods were analysed, together with the GPS and accelerometer data, using Catapult Sprint ver5.1.1 (Catapult Innovations, Canberra, Australia).

Player’s movements were determined with 10-Hz sample rate GPS units (MinimaxX S4, Catapult Sports, Canberra, Australia) during randomly selected training sessions encompassing all participants, at the beginning, middle, and at the end of the intervention period. A GPS unit was placed into a harness on the player’s upper back as described by the manufacturer. Maximal speed, total distance, distance covered at 0–2, 2–5, 5–9, 9–13, 13–16, 16–20 and > 20 km/h were measured. The speed zones were collated into low-speed movement (< 9 km/h), moderate-speed running (9–13 km/h) and high-speed running (> 13 km/h). Player Load (PL) and number of accelerations were measured using the accelerometers in the MinimaxX S4 at a 100-Hz sampling rate. PL is an estimate of physical demand combining the instantaneous rate of change in acceleration in three planes. PL is presented as time spent in PL Zone 0–0.1, 0.1–0.3, 0.3–0.6, 0.6–1.0, 1.0–1.5, 1.5–2.0, > 2.0 and total accumulated PL, whereas number of accelerations (measured using the accelerometer) is presented in totals (> 1.50 m/s/s) and in low (1.50–2.14 m/s/s), moderate (2.14–2.78 m/s/s) and high (> 2.78 m/s/s) accelerations in accordance with Randers et al. (2018a). The validity and reliability of the GPS units and accelerometers have been described by Boyd et al. (2011).

### Statistics

All statistical analyses were carried out with R Studio open-access software (R Core Team, Vienna, Austria). Data regarding intensity during training was analysed using students’ *t*-tests. Training intervention data were analysed using linear mixed models with time-group interactions (baseline/follow-up) as fixed effects and training attendance and baseline values as random effects. A visual inspection of residual plots and normal probability plots was used for model validation. To investigate the hypotheses of this study, specific sets of comparisons between and within groups were considered and analysed using global *F*-tests. Subsequently, linear mixed model-based t-tests were used for pairwise comparisons. Adjustment for a multiplicity of these pairwise comparisons was carried out using the ‘single-step’ adjustment, which achieved a less conservative adjustment than Bonferroni adjustment by utilizing correlations between tests. Data are reported as raw mean values with SD or changes as delta values with 95% confidence intervals unless otherwise stated. A significance level of 0.05 was applied. Effect sizes were calculated as the difference between the means divided by the pooled standard deviation and interpreted as suggested by Hopkins et al. (2009).

## Results

### Heart rate response and activity profile during training

**Fig. 1:**
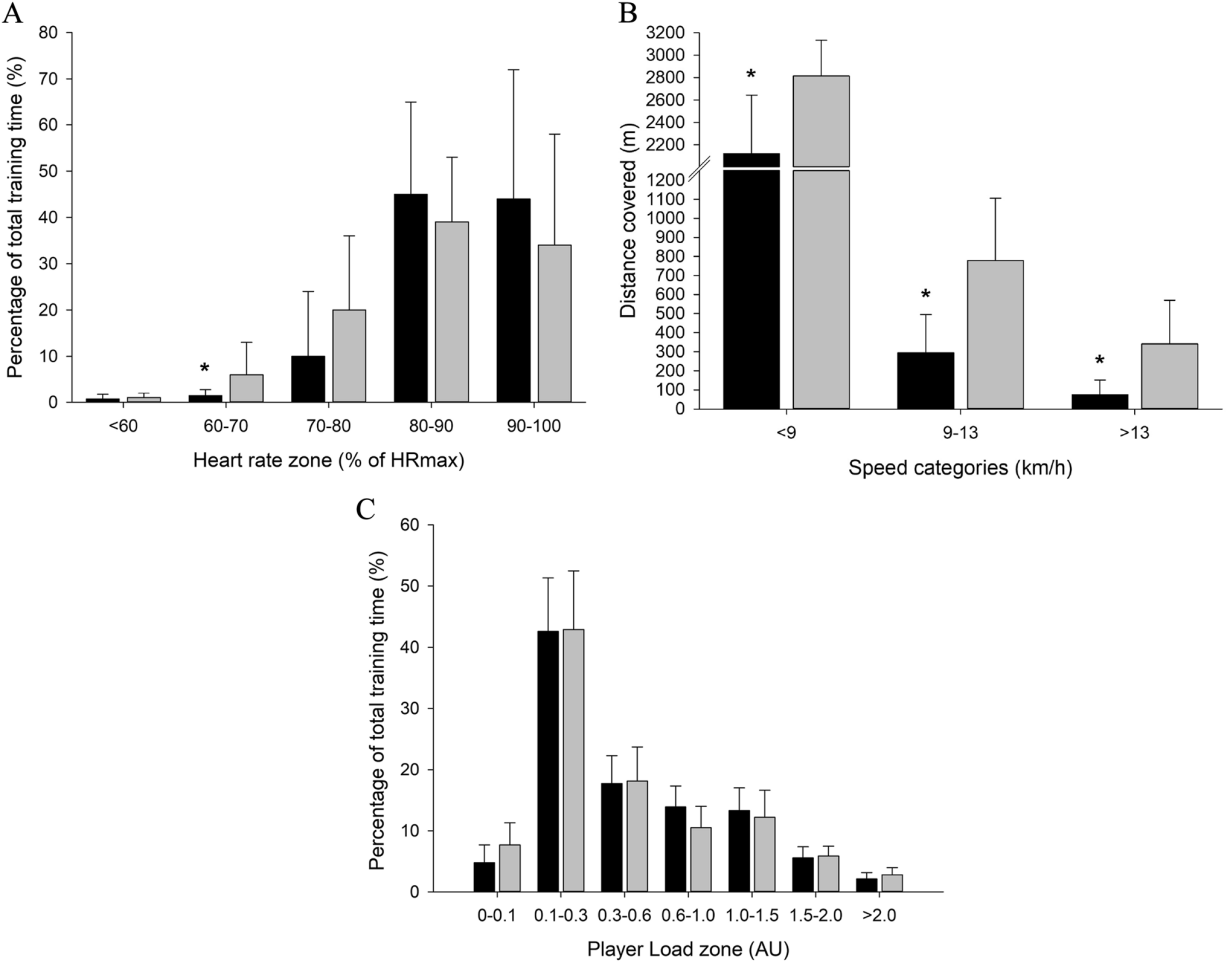
Intensity during training sessions described as **A** time spent in various heart rate zones, **B** distance covered in 3-speed categories, and **C** player load in various player load zones during street football training (ST; black bars) and grass football training (GR; grey bars). * Denotes significant (*P* < 0.05) difference from GR

Average and peak heart rate (HR) during the training sessions were high for ST as well as GR, with no significant differences between ST and GR in mean HR (87.1 ± 5.0 vs. 84.0 ± 5.3%HRmax; *P* > 0.05; ES = 0.60) nor in peak HR (97.4 ± 3.6 vs. 96.7 ± 3.3%HRmax; *P* > 0.50; ES = 0.20). HR distribution was not significantly different between ST and GR, with 44 ± 28 and 34 ± 24% of training time being spent above 90%HRmax for ST and GR, respectively (ES = 0.38; Fig. 1A).

Total distance covered was lower (*P* < 0.01) in ST than GR (2.69 ± 0.76 vs. 3.93 ± 0.84 km; ES = 1.55), corresponding to 56 ± 14 and 81 ± 17 m/min, respectively. Distance covered was lower (*P* < 0.05) in ST than in GR in all speed categories (Fig. 1B). Peak running speed was lower (*P* < 0.01) in ST than in GR (15.9 ± 2.8 vs. 19.7 ± 2.1 km/h; ES = 1.54). Total accumulated Player Load (PL) was not different between ST and GR (418 ± 84 vs. 411 ± 68 AU; *P* > 0.80; ES = 0.09), and no significant differences were observed in time spent in each PL zone (Fig. 1C). A higher number of accelerations was observed for ST compared to GR in total as well as in low, moderate, and high acceleration zones (547 ± 124 vs. 385 ± 126, ES = 1.30, 128 ± 54 vs. 78 ± 36, ES = 1.09, and 37 ± 17 vs. 21 ± 12, ES = 1.09, respectively; *P* < 0.05).

### Physiological response to treadmill running

**Fig. 2:**
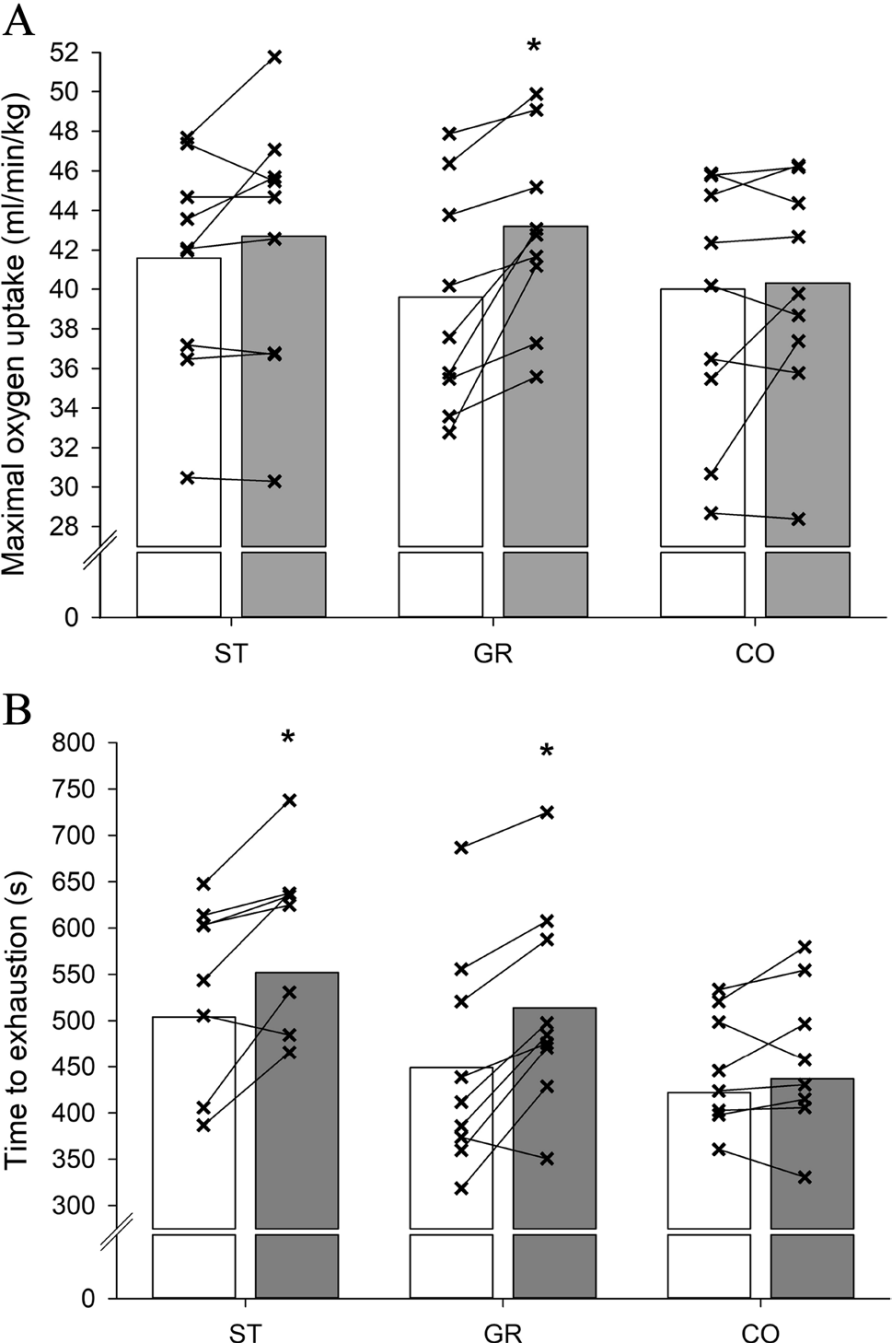
**A** Maximal oxygen uptake and **B** time to exhaustion before (white bars) and after (dark grey bars) the 12-week intervention with street football (ST) or grass football (GR) or an inactive control group (CO). *Denotes significant within-group difference (*P* < 0.05) and larger change than in CO

For maximal oxygen uptake, a time-group interaction (*P* < 0.05) was observed, and after 12 weeks of training, maximal oxygen uptake was elevated in GR (43.2 ± 5.2 vs. 39.6 ± 6.1 mL/min/kg; *P* < 0.001; ES = 0.64), but not (*P* > 0.05) in ST (42.7 ± 7.2 vs. 41.6 ± 6.1 mL/min/kg; ES = 0.16) or CO (40.3 ± 6.1 vs. 40.0 ± 6.1 mL/min/kg; ES = 0.05), with the change in GR (3.6 [1.8; 5.4] ml/min/kg) tending to be different from CO (0.3 [−1.0; 1.6] mL/min/kg, *P* = 0.079) and ST (1.1 [−0.4; 2.6] mL/min/kg, *P* = 0.085) (Fig. 2A).

A significant time-group interaction was found for treadmill time to exhaustion (TTE) (*P* < 0.05), and after 12 weeks of training, TTE was increased in GR (514 ± 110 vs. 449 ± 117 s; *P* < 0.001; ES = 0.57) and ST (552 ± 151 vs. 504 ± 137 s; *P* < 0.01; ES = 0.33) but not in CO (437 ± 97 vs. 422 ± 95 s; *P* > 0.05; ES = 0.16) with changes being higher in GR (64 [36; 93] s, *P* < 0.05) than CO (15 [−8; 37] s) (Fig. 2B).

For HR during submaximal running at 8 km/h, a significant time-group interaction was found (*P* < 0.001). HR during submaximal running at 8 km/h was lower after the training period in ST (141 ± 19 vs. 154 ± 21 bpm; *P* < 0.001; ES = 0.65) and GR (141 ± 10 vs. 157 ± 12 bpm; *P* < 0.001; ES = 1.45) but unchanged in CO (153 ± 15 vs. 155 ± 11 bpm; *P* > 0.60; ES = 0.15) with changes in ST tending to be (−14 [−17;-10] bpm, *P* = 0.083) and GR being (−12 [−19;−6] bpm, *P* < 0.05) larger compared to CO (−2 [2; −6] bpm).

A significant time-group interaction was found for blood lactate concentration after submaximal running at 8 km/h (*P* < 0.001), and blood lactate concentration was lower after the training period in ST (2.5 ± 1.3 vs. 4.5 ± 2.5 mmol/L; *P* < 0.001; ES = 1.00) and in GR (2.0 ± 1.0 vs. 2.7 ± 0.8 mmol/L; *P* < 0.001; ES = 0.77), but not in CO (3.2 ± 1.6 vs. 3.7 ± 1.4 mmol/L; *P* > 0.10; ES = 0.33) with changes being larger in ST (−2.0 [−2.8;−1.1] mmol/L) than GR (−0.7 [−0.9;−0.5] mmol/L, *P* < 0.001) and CO (−0.5 [−1.1;0.1] mmol/L, *P* < 0.01).

### Blood pressure and resting heart rate

No changes were observed in systolic blood pressure, diastolic blood pressure, mean arterial pressure or resting HR in ST, GR or CO after the 12-week intervention period (Table 1).

**Table 1:** Mean change and 95% CI for health and performance measures following a 12-week intervention with street football (ST) or grass football (GR) or an inactive control group (CO)

	ST			GR			CO		
	Mean	95% CI		Mean	95% CI		Mean	95% CI	
VO_2_max (mL/min)	85	−8	179	325	174	476	110	−14	234
Oxygen uptake during running 8 km/h (mL/min)	−157	−270	−44	58	−31	147	−41	−107	24
RER during running 8 km/h	−0.02	−0.04	0.01	−0.02	−0.05	0.02	0.00	−0.03	0.02
Blood lactate after running 8 km/h (mmol/L)	−2.0	−2.8	−1.1	−0.7	−0.9	−0.5	−0.5	−1.1	0.1
Heart rate during running 8 km/h (bpm)	−14	−17	−10	−12	−19	−5	−2	−6	2
Systolic blood pressure (mmHg)	1	−4	6	0	−1	2	−1	−4	3
Diastolic blood pressure (mmHg)	0	−2	2	0	−2	2	1	−1	4
Mean arterial pressure (mmHg)	0	−2	3	0	−1	2	1	−2	3
Resting HR (bpm)	0	−2	3	0	−4	4	−1	−5	3
Total body mass (kg)	−0.8	−2.2	0.7	−0.4	−2.4	1.6	−0.1	−1.0	0.7
Leg lean mass (kg)	0.5	0.1	0.9	0.4	0.0	0.7	−0.1	−0.3	0.2
Total lean mass (kg)	0.7	−0.5	2.0	0.4	−0.5	1.3	−0.3	−0.9	0.3
Bone mineral density (g/cm^2^)	0.004	−0.003	0.011	0.004	−0.016	0.024	−0.004	−0.012	0.005
T-score	0.0	−0.1	0.1	0.0	−0.2	0.3	0.0	−0.1	0.1
Body fat% (%point)	−1.5	−2.7	−0.2	−0.6	−1.6	0.4	0.2	−0.6	1.0
Android fat% (%point)	−1.1	−2.7	0.4	−0.6	−2.0	0.8	0.7	−1.1	2.4
Gynoid fat% (%point)	−2.0	−3.4	−0.5	−0.7	−2.3	0.9	0.0	−0.8	0.8
Jump length (cm)	3	−2	9	−6	−19	7	3	−8	14
Postural balance (number of falls)	−0.4	−4.4	3.6	−2.8	−4.8	−0.8	−3.3	−5.1	−1.5
30-m sprint time (s)	−0.16	−0.33	0.01	0.06	−0.15	0.28	0.19	0.07	0.31
Yo-Yo IE2 performance (m)	151	62	240	124	48	200	60	4	116

### Body composition

For body fat percentage, a tendency (*P* = 0.084) to a time-group interaction was found, and body fat percentage was decreased after 12 weeks in ST (28.3 ± 7.6 vs. 29.8 ± 7.2%; *P* < 0.05; ES = 0.20), but not in GR (29.7 ± 8.0 vs. 30.3 ± 8.5%; *P* > 0.50; ES = 0.07) and CO (28.6 ± 8.0 vs. 28.4 ± 8.2%; *P* > 0.90; ES = 0.02) with the change in ST (−1.5 [−2.7;−0.2]%) tending (*P* = 0.073) to be larger than CO (0.2 [−0.6;1.0]%) but not GR (−0.6 [−1.6;0.4]%, *P* > 0.40).

Total body mass, lean body mass, lean leg mass, whole-body bone mineral density (BMD) and T-score were unchanged in all three groups after the 12-week intervention period (Table 1).

### Blood analysis

No significant changes were observed in fasting blood glucose, plasma insulin, HbA1c, total cholesterol, LDL-C, HDL-C or triglycerides after the 12-week intervention period (Table 2).

**Table 2:** Mean change and 95% CI for blood measures following 12-weeks intervention with street football (ST) or grass football (GR) or an inactive control group (CO)

	ST			GR			CO		
	Mean	95% CI		Mean	95% CI		Mean	95% CI	
Glucose (mmol/L)	−0.1	−0.2	0.1	0.1	−0.1	0.3	0.2	−0.1	0.5
HbA1c (mmol/L)	0.0	−0.7	0.7	0.7	−0.7	2.1	0.5	−0.4	1.4
Insulin (pmol/L)	−1.2	−18.5	16.1	−13.3	−57.2	30.7	3.0	−9.1	15.1
Total cholesterol (mmol/L)	0.2	−0.1	0.6	−0.1	−0.4	0.2	−0.2	−0.4	0.1
HDL-C (mmol/L)	0.0	−0.1	0.1	0.0	−0.1	0.1	−0.1	−0.2	0.0
LDL-C (mmol/L)	0.2	−0.1	0.5	0.0	−0.2	0.3	0.0	−0.3	0.3
Triglycerides (mmol/L)	0.1	−0.2	0.3	0.0	−0.4	0.4	0.1	−0.2	0.3

*HbA1c* Glycosylated haemoglobin, *LDL-C *low-density lipoprotein cholesterol, *HDL-C *high-density lipoprotein cholesterol

### Performance tests

No significant changes were observed as a result of the intervention period in 30-m sprint time, postural balance, jump length or Yo-Yo IE2 performance in any of the groups (Table 1).

## Discussion

The main findings of the present study were that 12 weeks of 1–2 weekly 60-min street football training sessions led to improved submaximal exercise capacity but had no significant effects on maximal oxygen uptake or other health-related or performance variables, despite a high HR during the street football training sessions. For the small-sided grass football group, similar training effects were observed on submaximal exercise capacity and an additional positive and marked effect was observed in VO_2_max.

Surprisingly, VO_2_max was not significantly elevated in the street football group after 12 weeks of training (+1.1 mL/min/kg, *P* > 0.05), but only in the grass football group, showing a clinically relevant increase (+ 3.6 mL/min/kg corresponding to 9.1%, *P* < 0.05) (Aspenes et al. 2011; Nes et al. 2014). The magnitude of the increase in VO_2_max observed in the grass football training group is in line with changes observed in several other football training interventions (Krustrup et al. 2009, 2010b, 2018a; Randers et al. 2012; Milanović et al. 2015, 2019). It is somewhat surprising, that VO_2_max did not increase in the street football group as mean HR (84–87%HR_max_) and time above 90%HR_max_ (34–44% of playing time) were equally high in both groups during training. The intensity during the street football training was higher than typically reported during recreational small-sided football games (Krustrup et al. 2010a; Randers et al. 2010b, 2014b, 2018b) and street football for homeless men (Randers et al. 2012), but comparable to what has also been reported in 3v3 street football with boards for untrained healthy men (Randers et al. 2020). Thus, an increase in VO_2_max would have been expected for the street football group as periods with high intensity during football training have been suggested to be linked to improvements in VO_2_max and in cardiac function (Andersen et al. 2014).

The activity profile differed markedly between street and grass football training with ~ 50% more total distance covered, fourfold higher distance covered with high-speed and a higher peak running speed reached in grass football than street football, whereas 50% more accelerations were found in street football leading to a similar total accumulated Player Load. This different activity profile during street football is likely to include more static work than during grass football on larger pitches, which may have implications for the HR-VO_2_ relationship as HR increases more during light static exercise than would be expected from the increase in oxygen uptake (Hietanen 1984). The observed HR may therefore not reflect the oxygen uptake during very small-sided games and the aerobic load during the street football games may have been lower. Limited or no effects on VO_2_max have also been observed after 12–16 weeks of training with other team sports such as team handball and basketball played on very small pitches (~ 20 × 13 m) with comparable activity patterns and HR response (Hornstrup et al. 2020, 2018; Randers et al. 2018a). A recent study by Randers et al. (2020) that investigated the acute effects of very small-sided street football games showed, however, that energy turnover and oxidative stress were very high for short periods, as plasma ammonia and plasma uric acid increased markedly, even though running speed rarely exceeded 13 km/h, as also observed in the present study.

Training attendance in the present study (1.5–1.7 times per week) was on the lower end of that being reported in most previous football studies leading to an increase in VO_2_max of ~ 3.5 mL/min/kg (1.3–2.8 times per week) (Milanović et al. 2015). This shows, in accordance with Beato et al. (2017) that positive effects can be observed, at least after grass football training, even with lower training frequency than the biweekly recommendation (Krustrup and Krustrup 2018; Krustrup et al. 2018b). No correlations were observed between training attendance and changes in VO_2_max (*r* = 0.01–0.05), hence the slightly lower training attendance in the street football group is per se likely not an explanation for the lack of change VO_2_max. It may be speculated, whether a combination of low training frequency and total training volume combined with the activity pattern observed on very small-sided pitches may limit the effects on VO_2_max.

Exercise capacity was improved in both training groups as TTE in the incremental test was increased by 10–14%, and performance in the Yo-Yo IE2 test was improved by 25–29%, but the change was, however, only significantly different from the TTE in the grass football group. In the only previous study on the health effects of street football training, TTE was improved by 8% and Yo-Yo IE1 performance by 45% (Randers et al. 2012). Other indicators of improved exercise capacity are lowered HR and blood lactate during submaximal running, which in line with previous studies (Milanović et al. 2019), were observed in both training groups in the present study. The large drop in HR during submaximal running observed in ST and GR indicates an increased stroke volume. An increased stroke volume would, however, also be expected to be expressed as lowered resting HR, but no change in resting HR was observed in the present study in contrast with previous studies on recreational football training, in which reductions of 4 to 6 beats per minute were observed (Krustrup et al. 2010b, 2013; Andersen et al. 2010; Milanović et al. 2019).

In the present study, we did not find any changes in systolic or diastolic blood pressure. Nor was any change observed in blood pressure when analysing participants with elevated baseline values (systolic blood pressure > 125 mmHg and/or diastolic blood pressure > 85 mmHg). Changes in blood pressure are a common finding after 12–52 weeks of recreational football played as 5v5 to 9v9 on larger grass pitches (Krustrup et al. 2009, 2013, 2017; Beato et al. 2017; Mohr et al. 2014). As discussed above, the activity profile during very small-sided street football includes an increased static exercise load, which possibly affects blood pressure during exercise differently than during larger-sided grass football. In line with the findings on VO_2_max, team sports training using very small-sided games have no effect on blood pressure (Randers et al. 2012, 2018a; Hornstrup et al. 2018).

## Conclusion

The present study revealed that small-sided street football training elicits high heart rates and incorporates multiple intense actions for habitually active men, and that a training period of 12 weeks with 1–2 weekly sessions led to improvements in submaximal exercise capacity only, whereas the positive and marked training effects of recreational grass football on cardiorespiratory fitness was confirmed. Thus, governing bodies of recreational football for health should be aware of how training is organized as this may well influence the health outcomes.

## Data Availability

The datasets generated during and/or analysed during the current study are available from the corresponding author on reasonable request.
